# Characterization of a Fiber Bundle-Based Real-Time Ultrasound/Photoacoustic Imaging System and Its In Vivo Functional Imaging Applications

**DOI:** 10.3390/mi10120820

**Published:** 2019-11-27

**Authors:** He Leng, Yuhling Wang, De-Fu Jhang, Tsung-Sheng Chu, Chia-Hui Tsao, Chia-Hua Tsai, Steven Giamundo, You-Yin Chen, Kuang-Wen Liao, Chiung-Cheng Chuang, Tzong-Rong Ger, Li-Tzong Chen, Lun-De Liao

**Affiliations:** 1 Institute of Biomedical Engineering and Nanomedicine, National Health Research Institutes, Zhunan Township, Miaoli County 35053, Taiwan; herbleng1209@gmail.com (H.L.); y217834@gmail.com (D.-F.J.); tsaochiahui@nhri.org.tw (C.-H.T.);; 2 Department of Biomedical Engineering, College of Engineering, Chung Yuan Christian University, Chung Li District, Taoyuan City 32023, Taiwan; cheng965@cycu.edu.tw (C.-C.C.);; 3 Fiberoptics Technology Inc., Pomfret, CT 06258, USA; sgiamundo@fiberoptix.com; 4 Department of Biomedical Engineering, National Yang Ming University, Taipei 112, Taiwan; irradiance@so-net.net.tw; 5 Department of Biological Science and Technology, National Chiao Tung University, Hsinchu 300, Taiwan; liaonms@pchome.com.tw; 6 National Institute of Cancer Research, National Health Research Institutes, Zhunan Township, Miaoli County 35053, Taiwan; leochen@nhri.org.tw

**Keywords:** photoacoustic (PA), ultrasound (US), fiber bundle-based illumination, hemoglobin oxygenation saturation, in vivo imaging

## Abstract

Photoacoustic (PA) imaging is an attractive technology for imaging biological tissues because it can capture both functional and structural information with satisfactory spatial resolution. Current commercially available PA imaging systems are limited by their bulky size or inflexible user interface. We present a new handheld real-time ultrasound/photoacoustic imaging system (HARP) consisting of a detachable, high-numerical-aperture (NA) fiber bundle-based illumination system integrated with an array-based ultrasound (US) transducer and a data acquisition platform. In this system, different PA probes can be used for different imaging applications by switching the transducers and the corresponding jackets to combine the fiber pads and transducer into a single probe. The intuitive user interface is a completely programmable MATLAB-based platform. In vitro phantom experiments were conducted to test the imaging performance of the developed PA system. Furthermore, we demonstrated (1) in vivo brain vasculature imaging, (2) in vivo imaging of real-time stimulus-evoked cortical hemodynamic changes during forepaw electrical stimulation, and (3) in vivo imaging of real-time cerebral pharmacokinetics in rats using the developed PA system. The overall purpose of this design concept for a customizable US/PA imaging system is to help overcome the diverse challenges faced by medical researchers performing both preclinical and clinical PA studies.

## 1. Introduction

Neurophotonics has recently received significant attention in neuroscience because of its advantages of intrinsic contrast and nonionizing radiation [1]. However, pure optical imaging modalities [e.g., diffuse optical tomography (DOT)] often suffer from either insufficient spatial resolution or poor penetration depth [e.g., multiphoton microscopy, confocal microscopy, and optical coherence tomography (OCT)] due to strong light scattering. The penetration depth is extended to a few centimeters when using DOT with model-based reconstruction because DOT uses diffusive photons and is able to provide both optical scattering and absorption parameters [1]. However, poor spatial resolution (typically 1/5-th of the imaging depth) remains a problem with the DOT technique. A limitation of optical microscopy using ballistic or quasiballistic photons is that the maximum penetration depth is typically one optical transport mean free path (~1 mm). Because of this fundamental problem of light diffusion, pure optical imaging techniques have failed to realize widespread preclinical or clinical applications.

Photoacoustic (PA) imaging is an optical absorption-based hybrid imaging technology that combines intrinsic optical absorption with ultrasound (US) detection to provide multiscale spatial resolution and deep tissue penetration [2]. Optical absorption by biological tissues induces PA waves via the thermoelastic effect triggered when a pulsed laser in the visible or near-infrared (NIR) range is used to irradiate biological samples [3]. The optical absorption distribution in a sample can then be reconstructed using the PA signals detected by US transducers [2]. Other optical imaging modalities, such as OCT or DOT [4,5], also have this intrinsic contrast ability [6,7]. However, PA imaging provides additional advantages over these modalities, such as a deeper penetration depth (up to 5 cm) and higher contrast in biological tissue, thereby overcoming the inadequacies of pure optical imaging. Moreover, using intrinsic biological optical contrast (i.e., blood or melanin), this dual-modality US/PA imaging technique is able to offer both structural and functional information, such as information on angiogenesis, hemoglobin oxygen saturation and total hemoglobin concentration [8].

In view of this finding, a dark-field reflection-mode PA microscopic imaging technique using a high-frequency (i.e., >20-MHz) US transducer has been reported and has been demonstrated to be capable of tracking the in vivo blood oxygenation dynamics in a mouse brain undergoing global hypoxic or hyperoxic challenge [9,10]. Our previous studies have shown that functional photoacoustic microscopy (fPAM) using a 50-MHz ultrasound transducer is able to preclinically evaluate the in vivo changes in functional hemoglobin oxygen saturation (SO_2_) and cerebral blood volume (CBV) in the rat cortex in different disease models, such as ischemic stroke and transient ischemic attack [11]. Preclinical PAM applications have gradually expanded recently. In addition, researchers are focusing on translating PA technology from bench to bedside to make the unique clinical advantages of PA technology available for patient care. Potential clinical applications include breast cancer [12], sentinel lymph node (SLN) [13] and skin cancer [14] imaging. In clinical applications that require a large tomographic view, motorized stages can be used to control the position of the PA transducers in synchrony with the data acquisition sequence [1]. However, a gap in translating PA technology into a widely used technique for either preclinical or clinical imaging remains due to the extremely large size of the systems and the lack of a user-friendly interface [15], such as a truly programmable sequence in a single setting paired with a laser coupling system that can be easily set up for detection [1]. Another challenge in delivering high-energy laser pulses is that when focusing and coupling light into the fiber, the high peak power density can damage the surface of the fiber tip [16]. Thus, the energy that is output from the optical fiber is limited, restricting the illumination area and penetration depth of PA technology.

In this study, we established a new handheld dual-modality real-time ultrasound/photoacoustic imaging system (HARP). The developed HARP system is integrated with a fully programmable US system using MATLAB-based software, which can accommodate different types/frequencies of array transducers, allowing the user to choose transducers based on a specific application. Most importantly, a new concept using fiber bundles fixed to the transducer by a 3-D-printed jacket/adapter was employed to externally couple light energy to the imaging zone. Real-time US/PA images can be visualized using a MATLAB-based US platform, which performs all data acquisition, image processing and other tasks (e.g., switching between the laser and scanning stage). In vitro validation of the developed HARP system was performed at imaging depths up to 4 cm using different concentrations of blue and red inks to test the corresponding signal-to-noise ratios (SNRs) of the PA signals. We also demonstrated the performance of the developed HARP system for in vivo vascular mapping of both rat brain and rat tail veins. Finally, we tested the utility of the developed PA system for in vivo functional imaging, including its ability to image (1) in vivo stimulus-evoked cortical hemodynamic changes during forepaw electrical stimulation and (2) the pharmacokinetics of the contrast agent indocyanine green (ICG).

## 2. Materials and Methods

### 2.1. Handheld Dual-Modality Real-Time Ultrasound/Photoacoustic Imaging System with Fiber Bundle-Based Illumination

**Figure 1:**
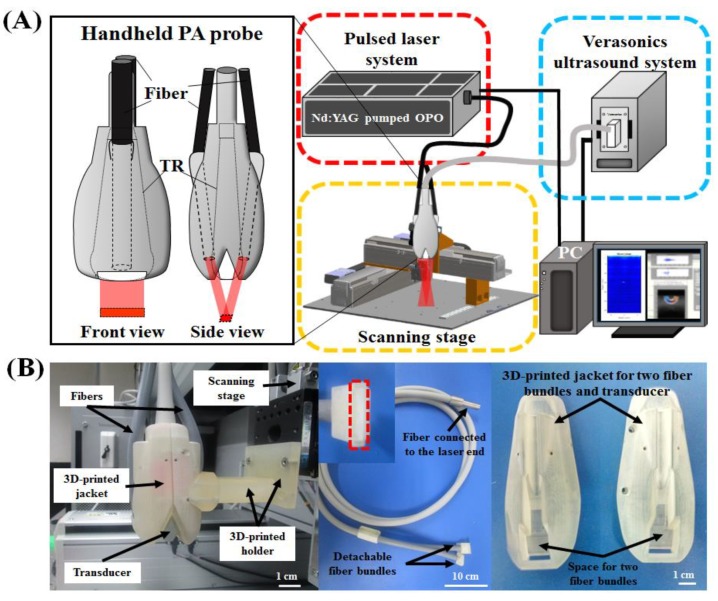
Schematic diagram of the developed handheld dual-modality US/PA imaging system with fiber bundle-based illumination. (**A**) Experimental setup of the developed PA system. A PC user interface including a control panel and program selection was integrated with a multichannel Verasonics US system and a pulsed laser system. (**B**) Photograph of the developed PA probe, which includes the removable fiber bundle-based illumination system (left), one US transducer, and a customized jacket (right). Photograph of the input and output ends of the fiber bundle (middle). The output end of the fiber bundle consisted of two rectangular parts, while the input end was circular. A zoomed-in section of the handheld PA probe fixed on an in-house scanning stage, showing the fiber jacket with the fibers and transducer, is displayed. US, ultrasound; PA, photoacoustic; Nd:YAG, neodymium-doped yttrium aluminum garnet; PC, personal computer; OPO, optical parametric oscillator; TR, ultrasound transducer.

**Figure 2:**
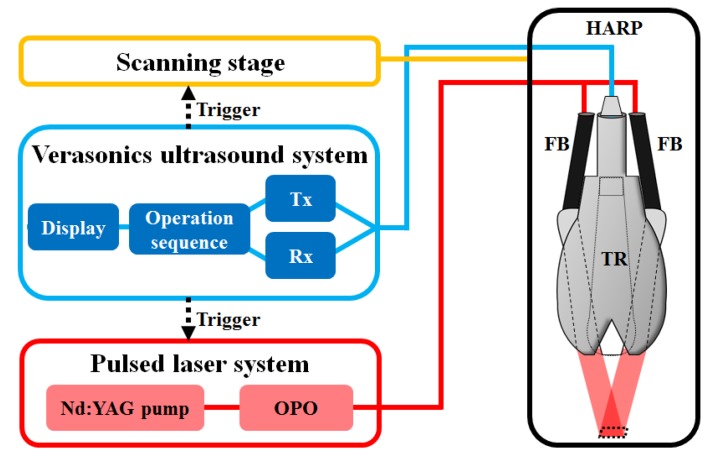
Operation sequence of the programmable dual-modality US/PA imaging system. The laser was pulsed with a pulse repetition rate of 20 Hz and coupled by a laser energy coupling system to a removable fiber bundle-based illumination system to illuminate the targets. PA waves were detected by an 18.5-MHz transducer and then processed through a Verasonics US system to the PC for further data analysis and display. US, ultrasound; PA, photoacoustic; HARP: handheld dual-modality real-time ultrasound/photoacoustic imaging system; TR, ultrasound transducer; FB, fiber bundle; OPO, optical parametric oscillator; Tx, transmitter; Rx, receiver.

The experimental setup of the developed HARP system is shown in Figure 1A, and the system block design is detailed in Figure 2. A 128-channel Verasonics high-frequency US platform (Vantage 128, Verasonics Inc., Washington, DC, USA) was employed for dual-modality imaging (both PA imaging and US imaging). The entire PA system was controlled by a custom-developed graphical user interface (GUI) based on MATLAB® (R2007a, MathWorks Inc., Natick, MA, USA). To operate the system in PA mode, laser excitation and data acquisition were synchronized using triggering. The PA signals were acquired by a high-frequency 18.5-MHz US transducer (L22-14v, Verasonics Inc., Washington, DC, USA). This transducer has a -6-dB fractional bandwidth of 67% and 128 active elements. The excitation laser was a compact Nd:YAG-laser system with an integrated tunable optical parametric oscillator (OPO, SpitLight 600 OPO, InnoLas Laser GmbH, Krailling, Germany). The OPO generates approximately 7-ns duration pulses at a 20-Hz repetition rate with tunable wavelengths from 680 to 2400 nm.

A homemade 3-dimensional (3-D) precision scanning stage (Figure 1A) was constructed such that the x- and y-axes are motorized by two piezoelectric motors (LMR (Linear Motor Robot), Toyo Automation Co., Ltd., Tainan City, Taiwan), and the z-axis can be manually adjusted with translation stages (Sigma-koki Co., Ltd., Tokyo, Japan); each step size is accurate up to 1 μm. This precision scanning stage is controlled by a PC-based program via the controller (PCI-1202U Driver Card, Advantech Co., Ltd., Taipei, Taiwan) and driver (ASD-A2R, Delta Electronics Inc., Taipei, Taiwan). That is, all parameters, including speed, acceleration and step size, can be easily set via the developed control interface. An optical ruler (RH200, Renishaw Inc., West Dundee, IL, USA), which is accurate up to 1 μm, was employed for positioning and providing the feedback signal. This 3-D precision scanning stage can provide accuracy for a moving step size as small as 1 μm, which is much smaller than the spatial resolution of US/PA imaging. The proposed PA system was developed to provide A-scan, B-scan (i.e., for two-dimensional (2-D) images where one axis is the lateral scanning distance and the other is the imaging depth), and C-scan (i.e., for 3-D images) images of the area of interest.

A diagram of the imaging operation sequence of the assembled PA probe is shown in Figure 2. During the imaging task, the developed PA probe was immersed in an acrylic water tank, which had a hole at the bottom sealed with a transparent 15-m-thick polyethylene film. The hole at the bottom served as an acoustic window. The imaging area was covered with a thin layer of US gel or a gelatin pad and attached to the thin film to ensure good coupling of PA waves. To synchronize the laser system, data acquisition by the Verasonics system, and the scanning stage, a trigger signal was transmitted at every laser illumination pulse. The received acoustic waves were reconstructed and displayed on the Verasonics computer screen at a frame rate of 20 frames per second.

### 2.2. Design of the Photoacoustic Probe—Integration of the Fiber Bundle-Based Illumination System, 3-D-Printed Jacket and Ultrasound Transducer

The customized fiber bundle-based illumination system (Fiberoptics Technology Inc., Pomfret, CT, USA) was designed to be 1.5 m long and to contain approximately 21,000 leaded glass fibers of 50-μm diameter with a high numerical aperture (NA). A schematic of this fiber bundle-based illumination concept for the proposed PA system is shown in Figure 1A,B. The fiber bundle was bifurcated at the distal end, allowing the light output to be delivered through two rectangular fiber arrays. The total area from which light is emitted was 16 × 3 mm, consistent with the active zone of the US transducer used. Each of these areas comprised 10,500 fiber tips. Each fiber had a core diameter of 47 μm (50 μm with cladding) with an NA of 0.66. By calculating the index of refraction (air and water), the manufacturer was able to control the incident laser illumination angle, so the laser output from the two fiber bundles was aligned approximately 4.5 cm beneath the surface of the transducer. 

A 3-D-printed jacket with two slots to hold the two output ends of the fiber bundle with appropriate light-emitting angles and a single slot to tightly accommodate the selected US transducer was designed. The entire PA probe (including the removable fiber bundle-based illumination system, one US transducer and a customized jacket) was attached to the three-dimensional scanning stage via a 3-D-printed holder. Both the jacket and holder were designed using the computer-aided design (CAD) software package SolidWorks 2015 (Dassault Systemes S.A., Waltham, MA, USA) and fabricated using a 3-D printer (ProJet 3500, 3D Systems, Inc., Rock Hill, SC, USA) with an accuracy of 0.016 mm. The incident angle of the fiber was optimized to 41° using the method from a previous study [17] to achieve coplanar light delivery and acoustic detection. The incident energy density on the imaging sample surface was estimated to be approximately 12 mJ/cm^2^, which is much lower than the American National Standards Institute (ANSI) safety limit (20 mJ/cm^2^) [18].

### 2.3. Performance Test of the Proposed Photoacoustic System

A transparent low-density polyethylene tube (Scientific Commodities, Inc., Lake Havasu, AZ, USA) with an inner diameter of 0.38 mm and an outer diameter of 1.09 mm containing blue ink (Lion Pencil Co., Ltd., New Taipei City, Taiwan) was placed in a water tank (270 mm × 180 mm × 128 mm) filled with 6% Intralipid (Sigma-Aldrich, Inc., Merck, Darmstadt, Germany) (optical absorption coefficient of 0.01 cm^−1^ and optical scattering coefficient of 400 cm^−1^) to mimic the optical diffusion in living biological tissues. Both the US and PA signal amplitudes were measured at various depths (0.5, 1.0, 2.0, 3.0 and 4.0 cm). The depths of the top and bottom surfaces of the tubes were measured in the US images. Signals were amplified, digitized and then passed through a bandpass filter before being stored in the Verasonics platform. In addition, the PA signal amplitudes from tubes containing various concentrations (undiluted and diluted 50%, 25% and 12.5% in saline) of blue ink were acquired at a depth of 9.6 mm with an excitation wavelength of 750 nm.

To assess spatial resolution, the developed PA system was also used for US/PA imaging of carbon fibers with a diameter of approximately 6 μm, as measured by a handheld light-emitting diode (LED) microscope (Aca1920-155um, Basler AG, Ahrensburg, Germany). A laser excitation wavelength of 750 nm was used to generate PA waves from the carbon fibers. Ten PA signals were acquired for each position and were then averaged to increase the SNR.

### 2.4. Photoacoustic Spectral Imaging of Blue and Red Inks

To understand the PA spectra of blue and red inks, we further recorded them in the NIR wavelength range in which the OPO operates. Blue or red ink (Lion Pencil Co., Ltd., New Taipei, Taiwan) was first injected into a transparent low-density polyethylene tube (Scientific Commodities, Inc., Lake Havasu City, AZ, USA) with a 0.38-mm inner diameter and 1.09-mm outer diameter. The tubes were then placed in a water tank filled with 6% Intralipid. The 18.5-MHz PA probe was used to record PA signals. Two laser excitation wavelengths, 750 and 850 nm, were used to facilitate spectroscopic analysis of the blue and red inks, respectively. Ten PA signals were collected and averaged for each experimental condition. The peak-to-peak PA signal intensity was plotted against the wavelength. PA signals were normalized according to the built-in laser power. To acquire C-scan images of the in vitro ink-filled tubes, a homemade motorized 3-D scanning stage was used to move the developed PA probe along the elevational (y) direction.

### 2.5. In Vivo Rat Brain Structure and Functional Imaging (Rat Left-Forepaw Electrical Stimulation)

Five male Wistar rats (National Laboratory Animal Center, Taipei, Taiwan) weighing 250–350 g were used for rat brain and tail vein imaging experiments. All animals were housed in a controlled room (temperature of 24 ± 1 °C, relative humidity of 50–60%) with a 12-h light/dark schedule (light cycle beginning at 7 am) and free access to both food and water. All experimental procedures, animal care protocols and protocols requiring ethical oversight were performed in accordance with guidelines approved by the Institutional Animal Care and Use Committee of the National Health Research Institute (approved protocol number: NHRI-IACUC-105089-A).

The animals were initially anesthetized with 3% isoflurane (Bowlin Biotech Corp., Taipei, Taiwan) in oxygen. Anesthetized rats were mounted on a custom-made acrylic stereotaxic head holder, and the skin and muscle were cut away from the skull to expose the bregma, which was used as a landmark. The anteroposterior (AP) distance between the bregma and the interaural line was directly surveyed. The bregma is located 9.3 ± 0.12 mm (mean ± standard deviation) anterior to the interaural line [19,20]. Furthermore, a craniotomy was performed on each animal to create a bilateral cranial window of approximately 8 (horizontal) × 6 (vertical) mm in size using a high-speed drill. Figure 2 depicts how the animals’ heads were positioned in our PA system and on the scanning stage. For the following experiments, the interaural line and bregma were then used as references to position the animals’ heads in our HARP system without additional surgery [20]. After bregma positioning, a PA C-scan (i.e., 2-D scanning) was performed to search the brain sections for the locations of B-scan images. The C-scan was also used to ensure that the rat’s head was located in the active zone of the 18.5-MHz transducer used for imaging.

After the PA B-scan, the rat brain vasculature within the region of the bregma +1 mm was imaged. A pair of homemade needle electrodes was inserted, one under the skin of the rat’s left forepaw and the other in the muscle of the upper arm [20]. Electrical stimulation was applied to the left forepaw using an electrical stimulator (Model 2100, A-M Systems, Sequim, WA, USA) with a monophasic constant current at a frequency of 3 Hz. The pulse duration was 0.2 ms with an intensity of 5 mA [20]. The animals were then anesthetized with 1.5% isoflurane in oxygen during this forepaw-stimulation experiment [21]. These parameters are able to elicit statistically significant sustained changes in physiological parameters during forepaw stimulation relative to baseline according to previous studies [22]. Laser excitation wavelengths of 750, 800 and 850 nm were used to acquire the functional US and PA images. At the selected wavelengths, blood is predominantly an absorber, demonstrating strong optical absorption and thus guaranteeing that the detected PA signals correspond mainly to blood vessels. US images are represented in grayscale, whereas PA images are represented in blue or red. To accurately identify regions of interest (ROIs), a modified laboratory-built imaging system was used to overlay PA images onto images of coronal brain slices from the rat brain atlas [11,22]. The overlaid images were used to define the anatomical borderlines of the primary somatosensory cortex of the forepaw (S1FL) from images scanned at the bregma +1 mm [20]. PA imaging of blood vessels in the rat tail was performed after the rat brain structural and functional imaging experiments using the same animals/numbers.

### 2.6. In Vivo Functional Indocyanine Green-Based Pharmacokinetic Imaging

Another five male Wistar rats (National Laboratory Animal Center, Taipei, Taiwan) weighing 250–350 g were used, and all the animal procedures in this experiment were performed as described in the previous section. ICG is a U.S. Food and Drug Administration (FDA)-approved nontoxic organic dye used in medical diagnostic applications [2]. The peak optical absorption of ICG bound with plasma proteins (Daiichi Sankyo, Inc., Tokyo, Japan) is at a wavelength of approximately 810 nm [2]. ICG in phosphate-buffered saline (pH 7.4) was injected intravenously at a dose of 0.25 mL/100 g body weight, which produced an estimated ICG blood concentration of approximately 1 × 10^−5^ M [2]. Before and after the injection of ICG, a series of PA images were obtained at 2 min and 30 min, respectively, to dynamically and continuously monitor ICG uptake in the rat brain [23]. After all imaging experiments, the rat was sacrificed by carbon dioxide inhalation.

## 3. Results and Discussion

### 3.1. Performance of the Developed Fully Programmable Ultrasound and Photoacoustic Imaging System

A photograph of the developed real-time fully programmable US/PA imaging system is shown in Figure 1. This newly developed PA system provides a programmable MATLAB-based research platform to implement user-designed operation sequences (Figure 2). All operation parameters are compiled to a sequence file (i.e., *.mat) that can be directly loaded in MATLAB. As shown in Figure 1B, the current components of the designed PA probe (including the fibers, jacket and holder) are inexpensive, ergonomic, and easy to fabricate or fine-tune because they are designed as a single piece without any overhanging features, eliminating the need for the assembly of parts. A laser energy coupling system was employed to optimize the field of view, depth of field and active diameter of the combined fiber bundle while minimizing the risk of damaging the fiber input. In this application, the fiber input is susceptible to damage caused by focused laser energy heating the interstitial epoxy to the point of burning. The use of a beam expander allowed us to use inexpensive leaded glass fibers with a high NA. The energy efficiency at the input and output ends of the fiber bundle-based illumination system was approximately 70%, which, to the best of our knowledge, is the highest output energy efficiency using single-fiber delivery for PA imaging reported to date. With this increase in output energy, the PA system can either illuminate a larger area at the same fluence or provide deeper tissue penetration.

**Figure 3:**
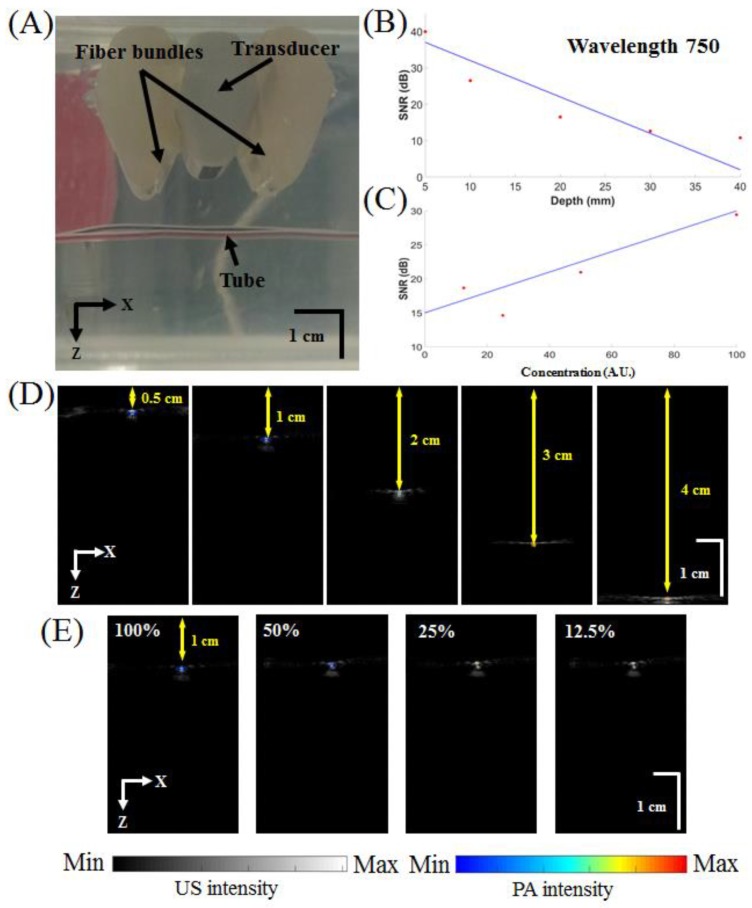
Performance test of the developed handheld dual-modality US/PA imaging system. (**A**) Photograph of the setup of the in vitro imaging experiment before the addition of Intralipid. (**B**) Quantified SNRs of blue ink at various depths (0.5, 1.0, 2.0, 3.0 and 4.0 cm) and (**C**) concentrations (undiluted and diluted to 50%, 25% and 12.5% in saline) of blue ink at an excitation wavelength of 750 nm. (**D**) Overlaid US/PA images at various depths. (**E**) Overlaid US/PA images for various blue ink concentrations (undiluted and diluted 50%, 25% and 12.5% in saline). US, ultrasound; PA, photoacoustic; A.U., arbitrary units; SNR, signal-to-noise ratio.

To test the in vitro performance of the developed PA system, the US and PA amplitudes of ink-containing tubes were measured at various depths and concentrations (Figure 3A–C). Figure 3D shows overlaid US/PA images of ink-containing tubes at various depths inside a water tank filled with 6% Intralipid. Figure 3E shows overlays of the tubes with various ink concentrations (undiluted and diluted 50%, 25% and 12.5% in saline) at a 1-cm depth. All PA images are depicted in pseudocolor with different dynamic ranges to represent the local optical absorption of the ink-filled tube, whereas all US images are depicted in grayscale with the same dynamic range. The US images were used to confirm that the PA signals originated from the tube. Figure 3B,C show the quantified SNRs of blue and red inks at various depths and concentrations. The SNRs of the PA signals from undiluted blue ink were 39.94, 26.48, 16.49, 12.62, and 10.78 dB at depths of 0.5, 1.0, 2.0, 3.0 and 4.0 cm, respectively. For ink concentrations of 12.5%, 25%, and 50% in saline and undiluted ink, the measured SNRs were 18.66, 14.62, 20.96, and 29.43 dB, respectively, at a fixed 1-cm depth. The SNR decreased linearly (10 dB/cm) with increasing depth (Figure 3B). The maximum detectable depth was approximately 4.1 cm, and the noise-equivalent depth was approximately 5.0 cm.

**Figure 4:**
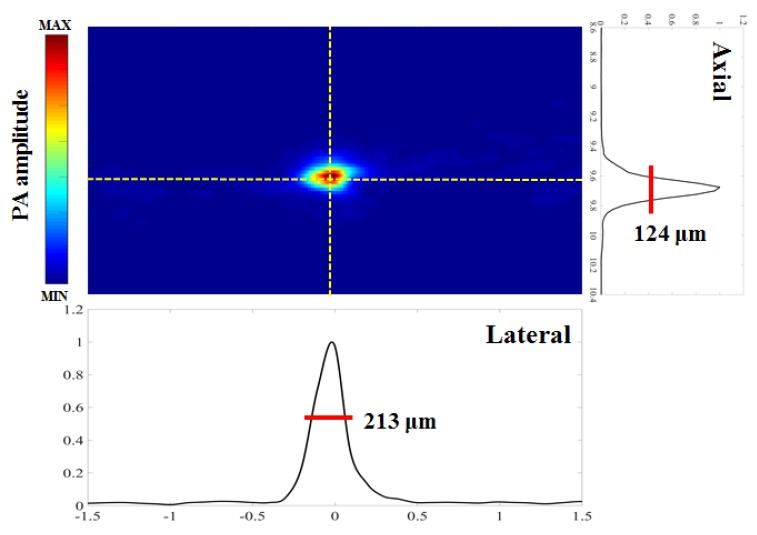
Axial and lateral resolution tests of the developed PA system at a depth of 9.6 mm quantified using a 6-µm carbon fiber and measured by full width at half maximum (FWHM). PA signals along the axial direction were normalized and fitted to a Gaussian distribution function. Despite the amplitude reduction with increasing depth, the axial resolution, which is calculated as the FWHM of each Gaussian function, remained at 124 ± 31 μm. PA, photoacoustic.

**Figure 5:**
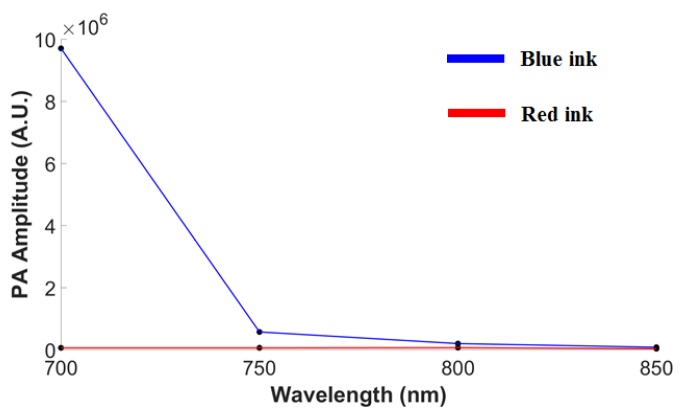
Quantified spectroscopic PA contrast of blue and red inks at excitation wavelengths of 700, 750, 800 and 850 nm. The PA amplitude values at wavelengths of 700, 750, 800 and 850 nm are 970.30, 57.10, 19.65, and 7.85 (×10^4^ A.U.) for blue ink and 5.91, 5.89, 6.55, and 3.17 (×10^4^ A.U.) for red ink, respectively. The difference between blue and red ink is highest when imaged at 700 nm, while the difference is not significant at 800 nm or above. PA, photoacoustic; A.U., arbitrary units.

Figure 4 shows axial resolutions at a depth of 9.6 mm, measured at the boundary of a 6-μm carbon fiber [24]. PA signals along the axial direction were normalized and fitted with a Gaussian distribution function. Despite the amplitude reduction with increasing depth, the axial resolution, which is calculated as the full width at half maximum (FWHM) of each Gaussian function, remained at 124 ± 31 μm. In addition, the spectroscopic PA contrast of the blue and red inks at varying wavelengths can be seen in Figure 5. The PA amplitude values at wavelengths of 700, 750, 800 and 850 nm are 970.30, 57.10, 19.65, and 7.85 (×10^4^ A.U.) for blue ink and 5.91, 5.89, 6.55, and 3.17 (×10^4^ A.U.) for red ink, respectively. Among these excitation wavelengths, the difference between blue and red ink is highest when imaged at 700 nm, while the difference is not significant at 800 nm or above.

### 3.2. In Vitro Ink-Filled Tube Phantom Imaging

**Figure 6:**
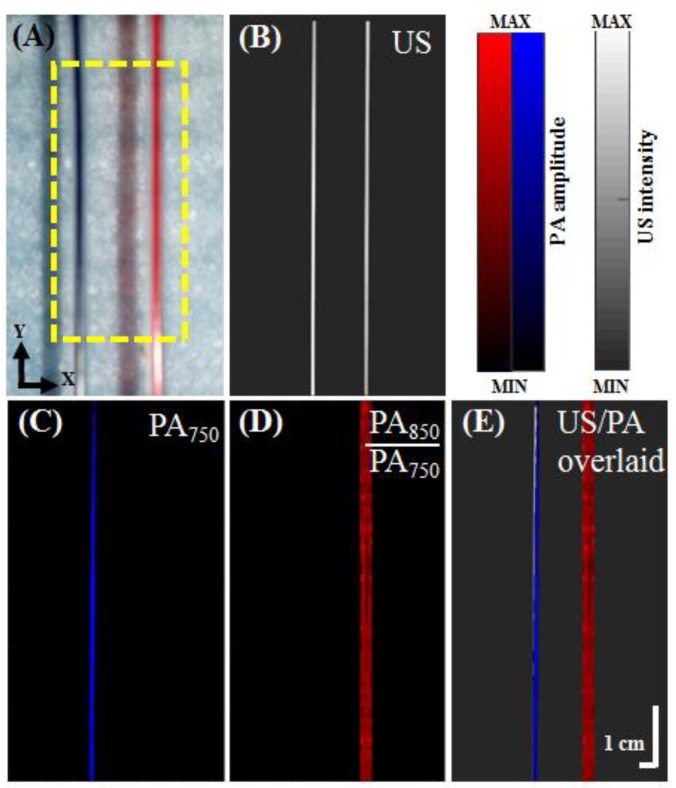
US/PA images of a vasculature-mimicking phantom. (**A**) Image of the phantom with a yellow dashed rectangle (6 mm × 12.695 mm) indicating the US/PA imaging region. (**B**) US MAP image of the phantom. (**C**) PA MAP image of the phantom at an excitation wavelength of 750 nm. (**D**) Proportional PA MAP image (PA850/PA750) of the phantom. The scanning step size and scanning speed were 0.1 mm and 2 mm/s, respectively, for panels B, C and D. (**E**) Overlaid US/PA MAP images of the phantom. US, ultrasound; PA, photoacoustic; PA750, PA signal at a 750-nm excitation wavelength; PA850, PA signal at an 850-nm excitation wavelength; MAP, maximum amplitude projection.

**Figure 7:**
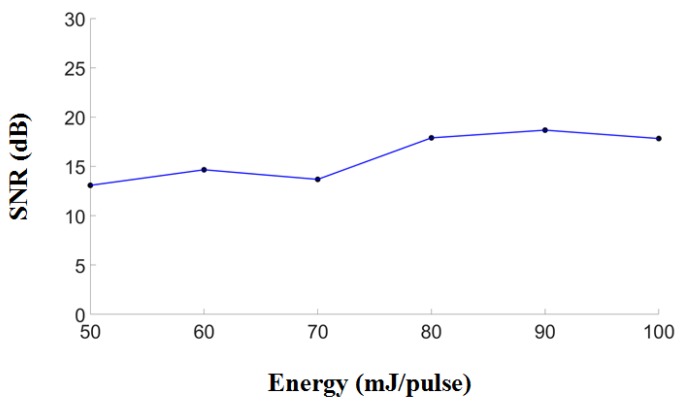
PA SNR as a function of the laser excitation energy. PA SNRs were acquired at different laser excitation energies and at a fixed depth of 1 cm and a fixed excitation wavelength of 750 nm for a 6-µm carbon fiber. The SNRs are 13.08, 14.66, 13.68, 17.89, 18.67, and 17.83 dB at energies of 50, 60, 70, 80, 90 and 100 mJ/pulse (directly measured from the laser output), respectively. The SNR improved slightly when the input laser pulse energy was increased from 50 to 100 mJ. PA, photoacoustic; SNR, signal-to-noise ratio.

To verify the volumetric imaging capability of the developed PA system, ink-filled tube phantoms were imaged. The results are shown in Figure 6A. To acquire a volumetric image, the PA probe was moved in the z-direction, and the planar images were combined. These volumetric images were then displayed in 2-D planes as maximum amplitude projections (MAPs). US images, PA images, and overlaid US/PA MAP images of the ink-filled tube phantom are shown in Figure 6B–E. In the US MAP images, the blue and red ink are indistinguishable (Figure 6B). The PA signals of the blue ink were dominant at 750 nm (Figure 6C), while the PA signals of the two inks were similar at 850 nm. However, the proportional difference (PARed = PA850/PA750) between the two excitation wavelengths (Figure 6D) allows us to distinguish the red ink from the blue ink. In addition, US and PA MAP images are overlaid in Figure 6E. The US images are presented in grayscale, whereas the PA images are presented in either blue or red scale. Both the optical absorption characteristics of the ink-filled tube phantom and structural information are shown in the resulting images. Figure 7 presents the PA SNR acquired at different excitation energies and at a fixed depth of 9.6 mm for the 6-µm carbon fiber. The SNRs are 13.08, 14.66, 13.68, 17.89, 18.67, and 17.83 dB at energies of 50, 60, 70, 80, 90 and 100 mJ/pulse (directly measured from the laser output), respectively. There was a slight increase in the SNR as the energy increased from 50–70 mJ/pulse, while the SNR remained stable between 80 and 100 mJ/pulse (directly measured from the laser output). 

### 3.3. In Vivo Vascular Imaging of Rat Brain and Tail

**Figure 8:**
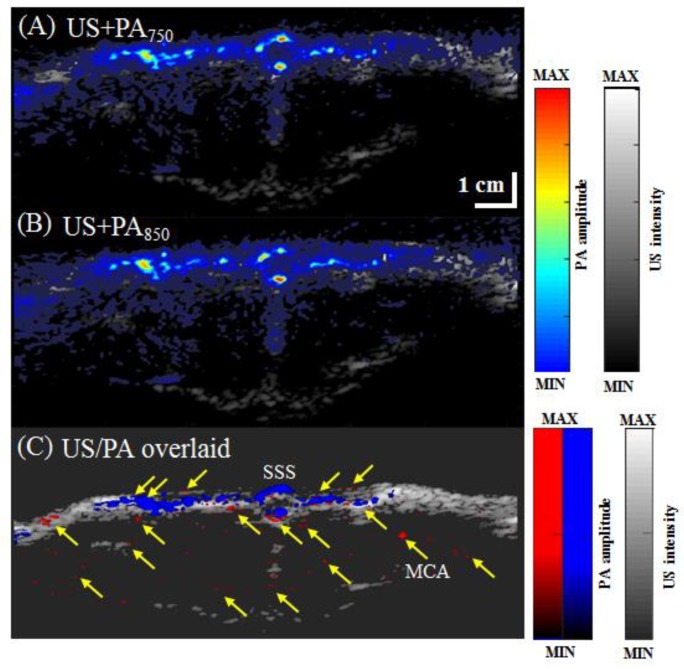
(**A**) In vivo US/PA B-scan images of a rat brain at +1 mm from the bregma, captured using the developed PA system. (**B**) US/PA B-scan images were acquired at excitation wavelengths of 750 nm and 850 nm. (**C**) Overlaid US/PA B-scan images of the rat brain. Solid yellow arrows indicate cerebral blood vessels. US, ultrasound; PA, photoacoustic; PA750, PA signal at a 750-nm excitation wavelength; PA850, PA signal at an 850-nm excitation wavelength; MCA, middle cerebral artery; SSS, superior sagittal sinus.

**Figure 9:**
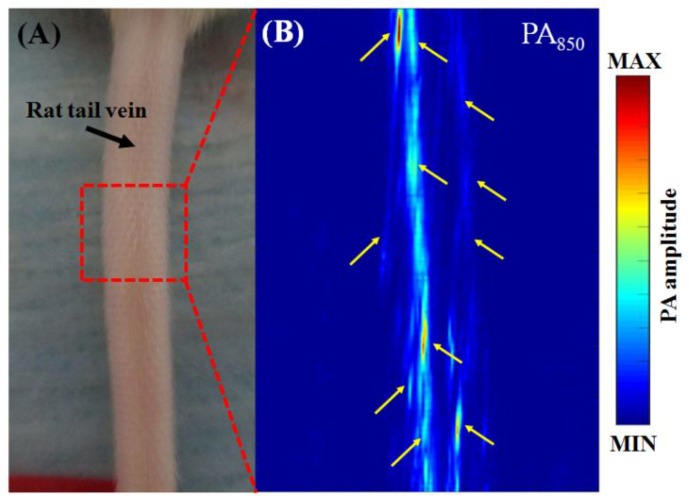
PA imaging of the microvasculature of a rat tail with intact skin. (**A**) Photograph of the in vivo rat tail. The red dashed rectangle (12.695 mm × 10 mm) marks the corresponding PA imaging region. (**B**) In vivo PA B-scan images of the rat tail microvasculature were acquired at an excitation wavelength of 850 nm. The solid yellow arrows indicate blood vessels of the tail. PA, photoacoustic.

To assess the application of our system for imaging living tissue, volumetric US/PA images of rat brain B-scan images and rat tail B- and C-scan images were acquired in vivo. Rat PA B-scan images of an open-skull window at wavelengths of 750 nm and 850 nm are shown in Figure 8A,B, respectively. Many blood vessels can be observed in these PA B-scan images. We can also visually observe the superior sagittal sinus (SSS) [25], branches from the middle cerebral artery (MCA) [25] and other relatively large blood vessels, as indicated by yellow solid arrows in Figure 8C. A PA C-scan of the rat tail vasculature at a wavelength of 850 nm is shown in Figure 9, where the tail veins are indicated by yellow solid arrows [26].

### 3.4. In Vivo Functional Imaging of Changes in Cortical Brain Hemodynamics during Left-Forepaw Electrical Stimulation

According to optical absorption spectral information found in the literature, the absorption at a wavelength of 850 nm is sensitive to changes in SO_2_, while the absorption at 750 nm is insensitive to changes in SO_2_ [27]. The absorption at a wavelength of 800 nm is sensitive to changes in CBV and insensitive to changes in SO_2_. PA imaging at 800 nm includes many other signals generated by different types of tissues, such as CBV. Therefore, to isolate changes in SO_2_ (e.g., excluding the effect of CBV), the PA images acquired at 750 or 850 nm were normalized to the PA images acquired at 800 nm, which served as a reference for CBV changes [20]. Therefore, independent measurement of the changes in SO_2_ and CBV can be achieved by the developed PA system, where the PA image at 800 nm is used as a marker of changes in the CBV and the PA image at 850 nm is used as a marker of changes in SO_2_ [28]. A movie of real-time PA imaging of changes in cortical brain hemodynamics during left-forepaw electrical stimulation is available online (Movie S1). 

**Figure 10:**
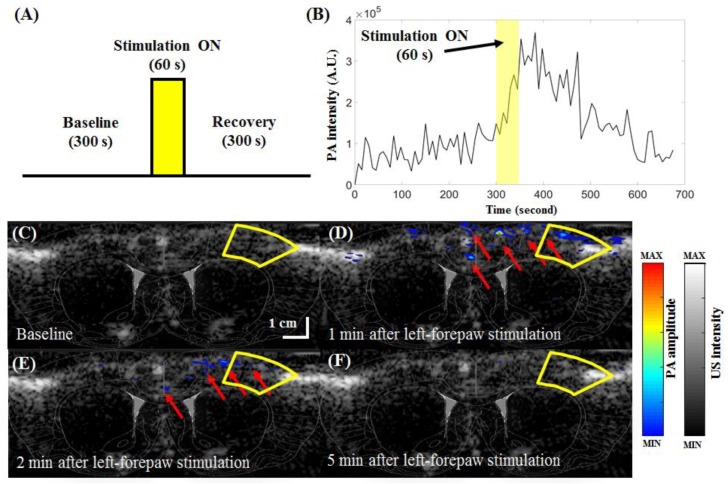
In vivo PA850 B-scan images of hemodynamic changes in the cortex of the rat brain at the position of bregma +1 mm (S1FL region) during left-forepaw electrical stimulation. (**A**) PA data collected in a block-design paradigm that utilized electrical stimulation of the left forepaw. The protocol began with 300 s in the baseline state. Then, a constant 3-Hz electrical pulse train with an intensity of 5 mA was delivered for 60 s during the active “Stimulation ON” state, which was followed by another 300-s period for recovery. PA B-scan imaging was started simultaneously with the baseline period and then stopped immediately at the end of the recovery period. PA B-scan images were acquired at an excitation wavelength of 850 nm. (**B**) Example plot of the PA signal from the S1FL region (outlined with solid yellow lines in (**C**–**F**)). Data from the baseline, stimulation ON (yellow rectangle) and recovery periods can be seen in the plot. The baseline intensity at t = 0 was subtracted from all PA signals. Baseline-subtracted US/PA-overlaid B-scan images at the baseline period and 1, 2, or 5 min after left-forepaw stimulation are shown in (**C**–**F**), respectively. Cerebral hemodynamics within a rat brain were also monitored in real time with 850-nm excitation (Movie S1). Significant differences in PA signals at 850 nm were observed in the contralateral S1FL ROI between the stimulation ON/OFF conditions. PA, photoacoustic; PA850, PA signal at an 850-nm excitation wavelength; S1FL, primary somatosensory cortex of the forepaw; US, ultrasound; ROI, region of interest; A.U., arbitrary units.

According to the designed stimulation protocol (Figure 10A), our forepaw electrical stimulation findings showed significant increases in SO_2_ (i.e., PA images at 850 nm) in the S1FL region at bregma +1 mm (Figure 10B–F). As shown in Figure 10B, the baseline intensity before stimulation was approximately 0.91 × 10^5^ A.U. in the S1FL region (outlined in solid yellow lines in Figure 10C–F). Immediately after stimulation, the intensity peaked at 3.69 × 10^5^ A.U. before decreasing to 1.29 × 10^5^ A.U., which was close to the baseline intensity. These results correlate well with the results from previous diffuse optical imaging (DOI) and functional magnetic resonance imaging (fMRI) studies [29], showing that SO_2_ changes on the side contralateral to stimulation are significantly higher than those on the ipsilateral side [5,30]. The present study newly shows that our US/PA system can directly measure changes in SO_2_ at 124 × 213 μm resolution to an imaging depth of up to approximately 4 cm, which cannot be reliably or directly detected using the other techniques mentioned herein [5,31]. In addition, conventional blood oxygenation level-dependent (BOLD) fMRI measures a mixed hemodynamic response [31], and isolating purely SO_2_ changes remains difficult [32]. Our data reflect activation-induced changes in PA850 (i.e., SO_2_) and further reveal that the strong BOLD responses detected in large draining veins may be mainly attributable to alterations in SO_2_ [33].

### 3.5. In Vivo Functional Imaging of Indocyanine Green-based Cerebral Pharmacokinetics in Rat

**Figure 11:**
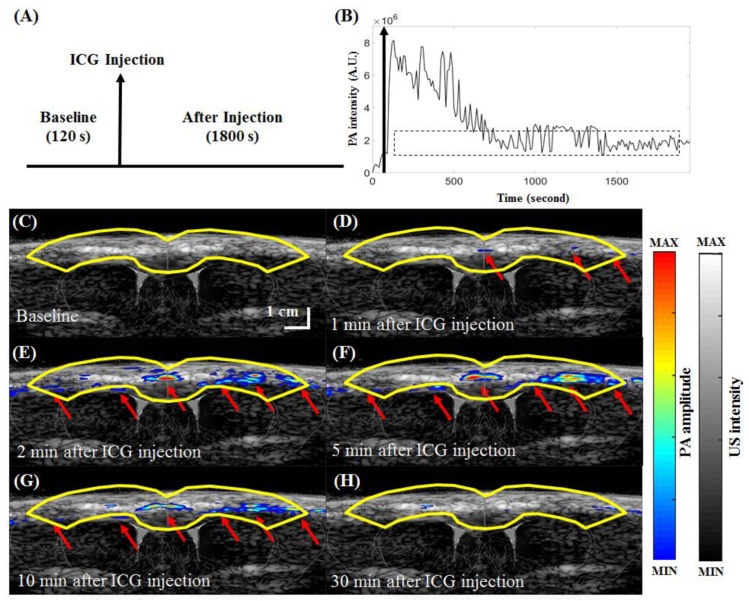
Real-time visualization of ICG pharmacokinetics acquired by the developed PA system. (**A**) US/PA data were collected in a block-design paradigm with ICG injection. The task began with 120 s in the baseline state. ICG was injected at the end of the baseline period. After injection, 1800 s of recovery time was monitored. US/PA B-scan imaging began simultaneously with the baseline period and was stopped immediately after the end of the recovery period. The PA B-scan images shown here were acquired at an excitation wavelength of 810 nm. (**B**) Example plot of the change in PA signal from the cortical region (outlined in solid yellow lines in (**C**–**H**)) with time. The baseline intensity at t = 0 was subtracted from all PA signals. ICG injection is indicated by the black arrow. Representative time frames of the baseline-subtracted US/PA overlaid B-scan images of the ROI in yellow at the time points of 5 min before and 1, 2, 5, 10 and 30 min after injection are shown in (**C**–**H**), respectively. Images were also combined into a video to illustrate the pharmacokinetics of ICG in real time (Movie S2). The PA images of the ICG-dyed brain acquired at the time points of 1, 2, 5 and 10 min after injection show that ICG accumulation enhanced the PA signal, as indicated by the red arrows, in the cortical region (yellow lines). ICG, indocyanine green; PA, photoacoustic; US, ultrasound; ROI, region of interest.

The ICG injection protocol for studying the in vivo functional imaging of ICG-based cerebral pharmacokinetics in rats is shown in Figure 11A, and the PA signals in the cortical region (outlined in solid yellow lines in Figure 11C–H) before and after ICG injection are shown in Figure 11B. Because of the half-life of ICG in the bloodstream and the constraints of linear array transducers, images were acquired in a coronal view. PA signals were recorded for 120 s before injection as a baseline and for another 1800 s after ICG injection. The peak signal intensity of 8.15 × 10^6^ A.U. was reached immediately after ICG injection. After approximately 800 s, the signal intensity in the cortex decreased to an average of 2.03 × 10^6^ A.U. Figure 11C–H show PA B-scan images of the cerebral ICG pharmacokinetics in rats 5 min before and 1, 2, 5, 10 and 30 min after injection. In PA images acquired at a wavelength of 810 nm, ICG contrast is dominant [2], and blood vessels are not detected [23]. That is, before the administration of ICG, there is no significant PA signal at a wavelength of 810 nm in the rat brain, as shown in Figure 11C. After the administration of ICG, contrast (PA imaging at a wavelength of 810 nm) is clearly visible through the cortical surface of the rat (Figure 11E–G). A movie of real-time PA imaging (at a wavelength of 810 nm) at various time points before and after ICG administration is available online (Movie S2). At 1800 s, there was no significant PA signal (at a wavelength of 810 nm) in the cortical regions of the rat brain, as shown in Figure 11H compared with Figure 11C (before ICG injection, baseline). Our data indicate that because the blood-brain barrier (BBB) remained intact, all the ICG in the cortical cerebrovascular system was automatically cleared by the body [34], which correlates well with the results of previous reports [23,35,36]. In Figure 11, the PA image is pseudocolored, while the US image is grayscale. The position and optical characteristics of the cortical layer corresponded well with the US/PA images.

In addition to the ICG-based PA imaging shown here, many other types of promising nanoparticles could also be used as contrast agents for in vivo PA imaging [37,38,39,40,41,42,43]. For example, gold nanoparticles (e.g., nanorods and nanocages), single-walled carbon nanotubes (SWNTs) and quantum dots (QDs) can all be detected using PA imaging [39,40]. ICG itself can also be encapsulated in different nanoparticle formulations for specific imaging applications [41]. An example is pH-sensitive ICG nanoparticles for characterizing the tumor microenvironment [41]. The developed HARP system has the potential not only to overcome the traditional depth and resolution limits of optical imaging but also to enable visualization of biological interactions through molecular imaging [42,43].

## 4. Conclusions

We developed a real-time hybrid US and PA imaging system, which consists of a detachable fiber bundle-based illumination system integrated with an array-based US platform. The energy efficiency at the input and output ends of the detachable fiber bundle-based illumination system was approximately 70%, which, to the best of our knowledge, is the highest output energy efficiency using single-fiber delivery for PA imaging reported to date. With this increase in output energy, our PA system can either illuminate a larger area at the same fluence or provide deeper tissue penetration while remaining within safety limits. The current PA system was successfully established for the in vivo detection of rat brain PA signals in response to changes in neuronal activity induced by forepaw electrical stimulation. Additionally, we demonstrated that the developed PA system was able to provide in vivo real-time cerebral pharmacokinetic mapping of the contrast agent ICG in the rat brain in a single setting. Thus, we showed that the in vivo measurement of cerebral hemodynamics/drug dynamics using a nonionizing and radiation-free instrument based on the intrinsic contrast of optical absorption is now feasible. Although the experiments in this study were performed with the probe inside a water tank, our US/PA imaging system can be easily adapted for handheld imaging applications using US gel. Overall, this customizable US/PA imaging system can complement existing optical imaging techniques and has the potential to be a useful tool for both preclinical and clinical PA studies.

## Supplementary Materials

The following are available online at https://www.mdpi.com/2072-666X/10/12/820/s1.
